# HIV-associated progressive multifocal leukoencephalopathy: longitudinal study of JC virus non-coding control region rearrangements and host immunity

**DOI:** 10.1007/s13365-013-0167-9

**Published:** 2013-05-29

**Authors:** Marco Iannetta, Anna Bellizzi, Sara Lo Menzo, Elena Anzivino, Alessandra D’Abramo, Alessandra Oliva, Claudia D’Agostino, Gabriella d’Ettorre, Valeria Pietropaolo, Vincenzo Vullo, Maria Rosa Ciardi

**Affiliations:** 1 Department of Public Health and Infectious Diseases, “Sapienza” University of Rome, Viale del Policlinico 155, 00161 Rome, Italy; 2 Department of Obstetrics, Gynecology and Urological Sciences, “Sapienza” University, Rome, Italy

**Keywords:** PML, JCV-NCCR, HIV, IRIS, Immune activation

## Abstract

John Cunningham virus (JCV), the etiological agent of progressive multifocal leukoencephalopathy (PML), contains a hyper-variable non-coding control region usually detected in urine of healthy individuals as archetype form and in the brain and cerebrospinal fluid (CSF) of PML patients as rearranged form. We report a case of HIV-related PML with clinical, immunological and virological data longitudinally collected. On admission (t0), after 8-week treatment with a rescue highly active antiretroviral therapy (HAART), the patient showed a CSF-JCV load of 16,732 gEq/ml, undetectable HIV-RNA and an increase of CD4+ cell count. Brain magnetic resonance imaging (MRI) showed PML-compatible lesions without contrast enhancement. We considered PML-immune reconstitution inflammatory syndrome as plausible because of the sudden onset of neurological symptoms after the effective HAART. An experimental JCV treatment with mefloquine and mirtazapine was added to steroid boli. Two weeks later (t1), motor function worsened and MRI showed expanded lesions with cytotoxic oedema. CSF JCV-DNA increased (26,263 gEq/ml) and JCV viremia was detected. After 4 weeks (t2), JCV was detected only in CSF (37,719 gEq/ml), and 8 weeks after admission (t3), JC viral load decreased in CSF and JCV viremia reappeared. The patient showed high level of immune activation both in peripheral blood and CSF. He died 4 weeks later. Considering disease progression, combined therapy failure and immune hyper-activation, we finally classified the case as classical PML. The archetype variant found in CSF at t0/t3 and a rearranged sequence detected at t1/t2 suggest that PML can develop from an archetype virus and that the appearance of rearranged genotypes contribute to faster disease progression.

## Introduction

Progressive multifocal leukoencephalopathy (PML) is a fatal brain demyelinating disorder caused by the human polyomavirus JC (JCV), resulting from lytic infection of oligodendrocytes (Padgett et al. 1971). In the 1980s, PML emerged as a major complication of HIV infection. The highly active antiretroviral therapy (HAART) era evidenced PML cases observed in patients with restored CD4^+^ T cells count, shortly after HAART initiation and defined as PML-immune reconstitution inflammatory syndrome (IRIS) (Falcò et al. 2008). In addition, PML cases which cannot be classified either as classic PML or as PML-IRIS are also reported (Mascarello et al. 2011). The increasing number of non-HIV/AIDS-related PML cases recently observed among patients treated with the immunomodulatory medications for autoimmune diseases, such as natalizumab, rituximab and efalizumab, highlighted the role of the immune system in the pathogenesis of PML (Bellizzi et al. 2012). JCV genome contains a well-conserved coding region and a hyper-variable non-coding control region (NCCR), which controls the early and late genes transcription and DNA replication. The well-conserved, non-pathogenic NCCR called *archetype* is most often detected in the kidney and urine of healthy individuals and immunosuppressed patients with or without PML (Yogo et al. 1991). Conversely, JCV NCCR rearranged variant showing duplications, tandem repeats, insertions and deletions is usually found in the blood, brain and cerebrospinal fluid (CSF) of PML individuals (Tan et al. 2010). After asymptomatic infection in childhood, JCV remains quiescent in the kidneys, bone marrow and lymphoid tissues. In the setting of immunosuppression, the virus may reactivate whereupon it can migrate into the brain, where genetic changes occur (neuroadaptation), allowing replication in the glial cells with PML development (White and Khalili 2011). Nevertheless, it is still debated whether JCV primary infection is triggered by archetype strain and NCCR rearrangement occurs during immunosuppression conditions (Fedele et al. 2003), or JCV rearranged form is required for initial infection in tonsil tissue (Sabath and Major 2002).

We report a case of JCV archetype-like variants-PML occurring in an HIV patient, shortly after a rescue HAART initiation which resulted in a persistent undetectable HIV viral load.

## Case report

A 51-year-old man with HIV-1 infection was admitted to our unit on March 2012 (t0) because of dysarthria and gait ataxia. HIV infection diagnosis was made in 2004, with CD4^+^ T lymphocytes nadïr of 4 cells/μl (1 %). The patient experienced multiple HAART failures, and HIV genotyping test showed a subtype B virus, with resistance mutations for protease inhibitors, nucleoside/nucleotide reverse transcriptase inhibitors, non-nucleoside reverse transcriptase inhibitors, integrase inhibitors and a CXCR4 tropism. On January 2012, 2 months before the onset of neurological symptoms, HIV-RNA was 2,527 copies/ml and CD4^+^ count was 9 cell/μl (2 %); a rescue HAART with ritonavir-boosted tipranavir, raltegravir, enfuvirtide and tenofovir/emtricitabine was started, and after 1 month treatment, HIV-RNA was undetectable (<37 copies/ml) and CD4^+^ count increased to 23 cells/μl (3.8 %).

**Fig. 1:**
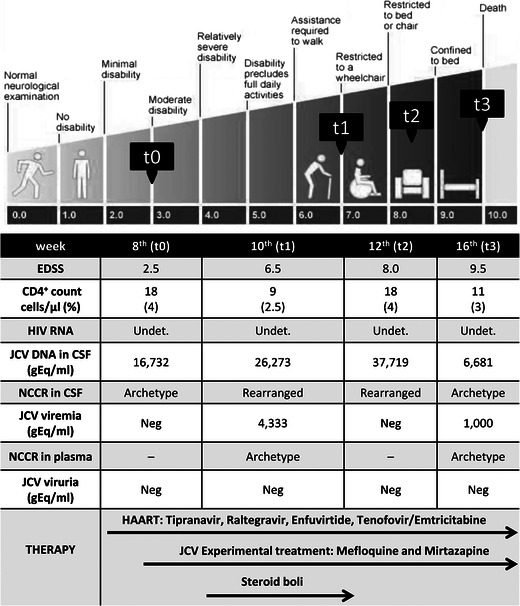
Clinical, immunological, virological and therapeutic history of the patient. The figure shows the clinical evolution we observed in our patient, from admission to death, according to the EDSS scale. We reported also the virological and immunological findings during hospitalisation. The *arrows in the lower part* of the figure represent the duration of HAART, mirtazapine and mefloquine experimental therapy and steroid treatment. JCV Q-PCR was performed on cerebrospinal fluid (CSF), plasma and urine samples and the JCV load is indicated in equivalent genome (gram equivalent per millimetre). *Undet.*, HIV load <37 copies/ml in the CSF and plasma. *Neg*, negative. *t0*, 8 weeks after starting the rescue HAART (hospital admission); *t1*, 10 weeks after starting the rescue HAART (2 weeks after hospital admission); *t2*, 12 weeks after starting the rescue HAART (4 weeks after hospital admission); *t3*, 16 weeks after starting the rescue HAART (8 weeks after hospital admission)

**Fig. 2:**
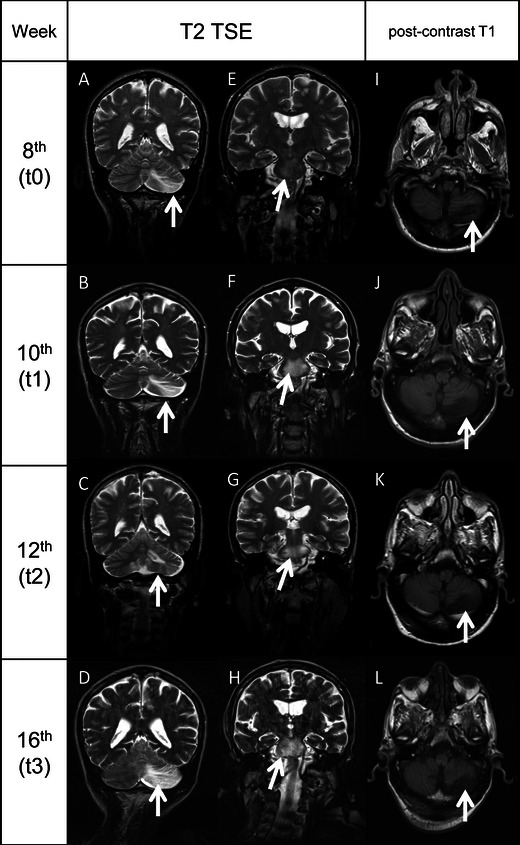
MRI evolution of the brain lesions. *A–D*, T2-TSE sequences showing the evolution of the left cerebellar lobe lesion (*arrows*) from t0 to t3. *E–H*, T2-TSE sequences showing the evolution of the lesion of the pons (*arrows*) from t0 to t3. No mass effect was seen in all the images collected. *I–L*, post-contrast T1-weighted sequences demonstrating the absence of contrast enhancement (*arrows*) in the left cerebellar lobe lesion (MRI). All the white matter lesions did not show enhancement in T1-weighted sequences after contrast administration. The lack of mass effect and contrast enhancement in all the MRI performed from t0 to t3 reduced the reliability of an IRIS-PML, which is usually characterised by a strong inflammatory infiltration of tissues, determining an alteration of blood–brain barrier which translates in penetration of contrast inside the lesions and oedema, which is the cause of mass effect

On admission (t0), physical examination showed a positive Romberg’s sign, dysarthria, gait ataxia and a Kurtzke Expanded Disability Status Scale (EDSS) score of 2.5. HIV-RNA was still undetectable and CD4^+^ count was 18 cells/μl (4 %) (Fig. 1). Brain magnetic resonance imaging (MRI) showed multiple hyperintense white matter lesions in T2-weighted turbo spin echo (TSE) sequences and fluid-attenuated inversion recovery imaging, in the temporal, cerebellar and pontobulbar regions. Diffusion-weighted imaging (DWI) and apparent diffusion coefficient (ADC) maps showed the presence of cytotoxic oedema. No contrast enhancement was seen in T1-weighted sequences (Fig. 2). CSF analysis showed normal cell count (2 cells/μl), normal levels of protein (39 mg/dl) and glucose (43 mg/dl) and normal results on cytological examination. CSF bacterial and fungal cultures and cryptococcal antigen were negative as well as herpes viruses assessed by CSF polymerase chain reaction (PCR). CSF HIV-RNA was undetectable (<37 copies/ml), whereas CSF quantitative JCV-DNA was 16,732 gEq/ml. PML was diagnosed and, suspecting IRIS, 5 days with intravenous (IV) methylprednisolone (1 g/day) followed by 8-week IV methylprednisolone tapered was added to HAART. Furthermore, after written informed consent, mefloquine 250 mg (guidelines of mefloquine treatment protocol, Biogen Idec 2012) and mirtazapine 30 mg (once daily) were administered to our patient, considering the mefloquine inhibitory effect on JCV replication in vitro and the mirtazapine activity in blocking type 2A serotonin cellular receptor for JCV entry in oligodendrocytes (Marshall and Major 2010). Clinical, neuroradiological, virological and immunological parameters were assessed in a longitudinal monitoring at 2 (t1), 4 (t2) and 8 (t3) weeks after admission.

Clinical evaluation at t1 showed worsened motor function, with difficulties in walking, requiring bilateral aid (EDSS score 6.5). At t2, dysarthria deteriorated and patient developed dysphagia and was confined to a wheelchair (EDSS was 8). At t3, the patient was aphasic and bedridden and a nasogastric tube was placed (EDSS was 9.5). Twelve weeks after admission, he died because of pulmonary oedema (Fig. 1).

MRI performed at t1, t2 and t3 showed a progressive extension of the lesions previously described, with cytotoxic oedema on DWI sequences and ADC maps. There was no mass effect or contrast enhancement in all the T1-weighted images performed (Fig. 2).

HIV viral load was persistently undetectable in plasma and CSF samples. Conversely, a JC viral load of 26,263 gEq/ml in the CSF and 4,333 gEq/ml in the plasma was found at t1 (Fig. 1). At t2, CSF JCV load increased up to 37,719 gEq/ml whilst plasma resulted negative. Finally at t3, the CSF JC viral load decreased to 6,681 gEq/ml and JCV-DNA reappeared in plasma with 1,000 gEq/ml. In all urine samples, JCV was persistently negative (Fig. 1).

**Fig. 3:**
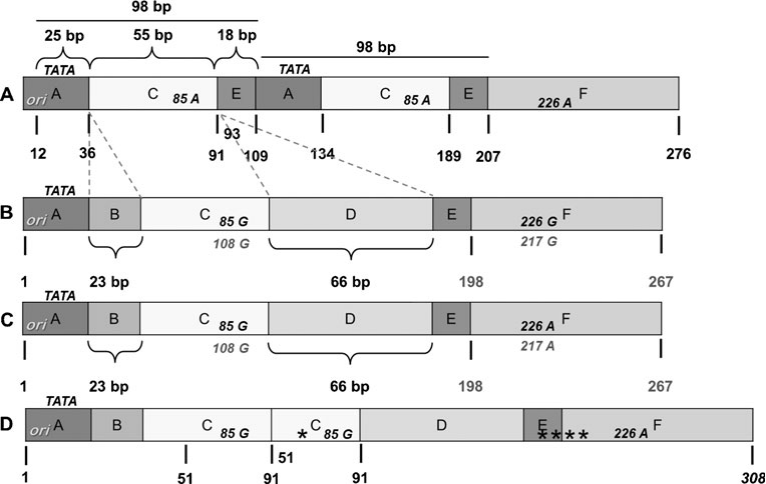
PML-associated variant Mad-1 (*A*), archetype CY (*B*) and JCV NCCR structures found in samples (*C*, *D*). The nucleotide sequences are shown from the core of the origin of DNA replication (ori) to the start site of the late leader protein, Agno protein. In *A*, the nucleotide numbering is based on PML-associated variant Mad-1 NCCR sequence and numbers are indicated in *bold* and *black*. In *B*, the nucleotide numbering of the archetype sequence of Japanese strain CY, isolated by Yogo et al. (1991) is reported in *bold* and *grey*. In *C*, the archetype variant sequence found in CSF specimens collected at t0 and t3, in plasma at t1 and t3 and in PBMC at t0, t1 and t3 is reported. In *D*, the rearranged JCV NCCR sequence of 308 bp in length, obtained from the CSF samples collected at t1and t2, is showed. *Asterisks* represent single nucleotide point mutations or deletions. The TATA box is presented by *TATA*. *Italicised capital letters* indicate nucleotides. Mad-1 NCCR contains an adenine at positions 85 and 183, compared with archetype CY NCCR, which contains guanine at these positions. Boxes division from A to F is also shown in *capital letters*. The sequence analysis was performed directly on DNA template previously amplified by nested-PCR, using two pairs of primers that anneal to the invariant regions flanking the NCCR of JCV. Primers BKTT1 (5′-AAG GTC CAT GAG CTC CAT GGA TTC TTC C-3′) and BKTT2 (5′-CTA GGT CCC CCA AAA GTG CTA GAGCAG C-3′) amplified a 724-bp DNA fragment in JCV (Mad-1). The second pair, JC1 (5′-CCT CCA CGC CCT TAC TAC TTC TGA G-3′) and JC2 (5′-AGC CTG GTG ACA AGC CAA AAC AGC TCT-3′), amplified a portion of the first round PCR product, generating a fragment of 308 bp (Pietropaolo et al. 2003)

JCV NCCR sequence analysis was performed in all samples as well as in peripheral blood mononuclear cells (PBMC) by real-time quantitative PCR (Q-PCR). Sequencing of PCR products was directly performed by using primers previously reported (Pietropaolo et al. 2003). JCV archetype variant was found in CSF specimens collected at t0 and t3, in plasma at t1 and t3 and in PBMCs at t0, t1 and t3. CSF samples collected at t1 and t2 showed a JCV NCCR rearranged sequence characterised by a duplication of the box C, containing the *cre*-*TAR* binding site for the HIV-tat protein (Fig. 3). In addition, JCV subtype 1B was found in all JCV-DNA-positive samples, performing direct sequencing of a 215-bp fragment amplified from the major capsid protein (VP1) gene (Agostini et al. 2001).

Our patient had a very low CD4^+^ cell count in peripheral blood, with CD4/CD8 ratio ranging from 0.04 to 0.05. The same finding was observed in CSF, with a CD4/CD8 ratio ranging from 0.12 to 0.22. During follow-up, high levels of immune activation (defined by flow cytometry as percentage of double positive HLA-DR and CD38) were observed for CD4+ and CD8+ T lymphocytes, both in peripheral blood and CSF samples.

## Discussion

Our patient developed PML after 2 months of an effective rescue HAART, resulting in undetectable HIV-RNA and initial rise of CD4^+^ cell count (100 % increase). In this scenario, the sudden onset of neurological symptoms and signs shortly after HAART, without other cerebral disorder, led us to consider PML-IRIS as plausible (Cinque et al. 2009; Gheuens et al. 2012; Harrison et al. 2011). Although MRI performed four times did not show brain lesions enhancement in T1 sequences after contrast administration (a strong suggestion of blood–brain barrier disruption and inflammation indicating IRIS), PML-IRIS was still considered, based on a retrospective analysis showing MRI contrast enhancement of brain PML lesions occurring only in 56.7 % of PML-IRIS cases (Tan et al. 2009). Hence, an experimental JCV combined treatment with mefloquine and mirtazapine was added to steroid boli but failed to alter disease progression. CSF JCV viral load remained persistently elevated and JCV remained detectable in the plasma.

T cell activation seems to be the primary characteristic that defines pathogenic versus non-pathogenic SIV infection in non-human primates models (Silvestri et al. 2007). Among untreated HIV+ patients, T cell activation has been found as an independent predictor of disease progression (Giorgi et al. 2002). In treated subjects, T cell activation is associated with lower CD4^+^ T cell gains during treatment (Hunt et al. 2003). During suppressive HAART, high level of immune activation is predictive of mortality due to AIDS- and non-AIDS-defining clinical events (Butler et al. 2011).

In our patient, high level of CD4^+^ and CD8^+^ T lymphocyte immune activation were persistently observed. On these assessments, it was hard to classify this case as either classical PML or IRIS, therefore emphasising the need for new diagnostic tools to better differentiate the two PML forms. Despite that proton magnetic resonance spectroscopy has been recently reported as useful to identify PML-IRIS lesions by their metabolism (Gheuens et al. 2012), unfortunately we were unable to perform spectroscopy in our patient.

JCV NCCR rearranged form was detected in the CSF samples collected at t1 and t2 and it was characterised by a duplication of the box C containing the *cre*-TAR binding site for the HIV-*tat* protein. Tan et al. (2010) have also found a similar rearranged sequence and the JCV NCCR archetype in the brain of HIV-positive patients with PML. We directly performed the sequencing of PCR products, detecting only the main JCV variant produced by the lytic infection of brain cells at any given time. Therefore in our patient, we cannot conclude whether the rearrangements at times t1 and t2 may be generated from the archetypal variant found at t0 or more viral variants established latency in the glial cells. Nevertheless, our data showed the strict association between the presence of the JCV rearranged forms in CSF and the clinical worsening, confirming their role in PML pathogenesis.

Finally, the finding of the archetype variant, in association with a relative lower number of JC viral load in CSF at t0 and t3, requires a reassessment of the archetypal JCV strain role in the lytic infection of brain cells. In fact in a recent report, Dang et al. (2012) focused on a novel JCV variant, harbouring an archetype-like NCCR, in a patient affected by JCV encephalopathy. In our case, it seems reliable that PML came out as local brain reactivation of latent virus since a conserved NCCR archetype form was detected in the plasma and PBMC throughout the observation period.

It is noteworthy that the JCV rearranged variant detected in our patient showed a duplication of HIV-tat binding region, despite undetectable HIV-RNA in CSF sample. Viral proteins, such as *tat*, may be the results of transcription of both integrated and unintegrated HIV-DNA, and the latter could increase after the initiation of HAART containing an integrase inhibitor (Sloan and Wainberg 2011).

It is interesting to note that JCV-DNA was never detected in urine samples of our patient despite the severe immunosuppressed status. Studies specifically focused on the pathogenesis of PML suggest that JCV reactivation in the kidney may not be related to PML (Koralnik et al. 1999), and no association between JCV viruria and subject’s immunological status has been demonstrated (Marzocchetti et al. 2009). Finally, JCV subtype 1B, the predominant genotype in Southwest Europe (Agostini et al. 2001), was detected, suggesting JCV primary infection as acquired in Italy. In conclusion, this case suggests that when a severe HIV-related immune deficiency occurs, JCV archetypal strain harbouring the brain in a latent state may replicate causing brain white matter lesions resulting in PML. Hence, JCV NCCR rearrangement seems not required to trigger JCV brain infection but its occurrence may worsen the PML’s clinical course. More studies are needed to fulfil the comprehension of JCV archetype variant role in the PML pathogenesis and the factors regulating NCCR rearrangement in vivo.
